# Ultrafast room-temperature valley manipulation in silicon and diamond

**DOI:** 10.1038/s41567-025-02862-4

**Published:** 2025-04-14

**Authors:** Adam Gindl, Martin Čmel, František Trojánek, Petr Malý, Martin Kozák

**Affiliations:** Department of Chemical Physics and Optics, Faculty of Mathematics and Physics, Charles University, Prague, Czech Republic

**Keywords:** Electronic properties and materials, Ultrafast photonics

## Abstract

Some semiconductors have more than one degenerate minimum of the conduction band in their band structure. These minima—known as valleys—can be used for storing and processing information, if it is possible to generate a difference in their electron populations. However, to compete with conventional electronics, it is necessary to develop universal and fast methods for controlling and reading the valley quantum number of the electrons. Even though selective optical manipulation of electron populations in inequivalent valleys has been demonstrated in two-dimensional crystals with broken time-reversal symmetry, such control is highly desired in many technologically important semiconductor materials, including silicon and diamond. We demonstrate an ultrafast technique for the generation and read-out of a valley-polarized population of electrons in bulk semiconductors on subpicosecond timescales. The principle is based on the unidirectional intervalley scattering of electrons accelerated by an oscillating electric field of linearly polarized infrared femtosecond pulses. Our results are an advance in the development of potential room-temperature valleytronic devices operating at terahertz frequencies and compatible with contemporary silicon-based technology.

## Abstract (web summary)

Control over electron populations in different conduction band minima in semiconductors can be used to store and process information. Now the ultrafast optical manipulation of such electrons at room temperature has been demonstrated in silicon and diamond.

## Main

In crystalline materials, the electron wavefunction follows the periodicity of the lattice and can be written as a Bloch wave *ψ*_*n***k**_(**r**) = *u*_*n***k**_(**r**)e^i**k.r**^, where *u*_*n***k**_(**r**) is the spatially periodic part and **k** is the wavevector of the electron. The solution of the time-independent Schrödinger equation leads to electronic states in the form of continuous bands with energy dispersion *E*_*n*_(**k**), where *n* is the band index. Semiconductors and dielectric materials are characterized by having a band of forbidden energies separating the highest occupied valence band and the lowest unoccupied conduction band.

The dispersion relation *E*_c_(**k**) of the conduction band has either a single minimum in the centre of the first Brillouin zone (**k** = 0) or several energy-degenerate minima (valleys) corresponding to different values of the wavevector **k**. In materials with several conduction band valleys, the quantum number associated with the wavevector **k** of the occupied valley can be used for transporting, processing and storing information instead of the electric charge of the electron, which is exploited in classical electronics.

In specific two-dimensional crystals with broken time-reversal symmetry (for example, two-dimensional transition-metal dichalcogenides^1,2^), spin–orbit coupling leads to the existence of two inequivalent groups of energy-degenerate valleys with opposite electron spins^3^. The excitons can be populated to a specific group of valleys thanks to the selection rules for optical transitions induced by circularly polarized light^4–6^. The valley quantum number can be selected by the handedness of the circular polarization of resonant photons generating the excitons in these materials. However, in bulk semiconductors with several valleys, such as silicon or diamond, such selection rules do not exist due to the crystal symmetry. Alternative forms of manipulation for specific conduction band valleys are based on removing the energy degeneracy with a static electric or magnetic field^7–11^, by spatial confinement of the electrons^12^, by optical coherent phenomena^13–15^ or by a non-resonant strong-field interaction with shaped intense fields^16,17^.

A conceptually different approach has been developed to generate, transport and detect the valley-polarized population of electrons in diamond at low temperatures^18,19^. The approach is based on applying a strong static electric field along the [100] crystallographic direction. Owing to the anisotropic acceleration of electrons in inequivalent valleys caused by the anisotropy of the effective mass tensor, the intervalley electron–phonon scattering rate is higher when electrons scatter from a valley with a small effective mass in the direction of the applied field to a valley with a large effective mass. The populations of electrons from different valleys can be detected electrically by time-of-flight measurements of the electrical current and can be separated by applying a magnetic field in Hall effect measurements^18^. This method of achieving and detecting valley polarization requires the anisotropic electron distribution to persist in the crystal for several tens of nanoseconds because the charge transport must persist over macroscopic distances so that it can be collected by the electrodes. The valley-polarized electron population relaxes to an isotropic distribution in reciprocal space through intervalley electron–phonon scattering. The change in the wavevector of an electron Δ**k** during its transition to another valley is compensated for by the creation or annihilation of a phonon, which has wavevector **q** = −Δ**k**. Although the characteristic intervalley scattering time of electrons in semiconductors and dielectric crystals at cryogenic temperatures is up to 1 ms, it decreases to femtosecond to picosecond timescales at room temperature^20,21^. For this reason, the valley polarization of electrons has not been observed in bulk semiconductors or dielectric crystals at room temperature because it requires an ultrafast techniques, for both its generation and detection.

**Fig. 1:**
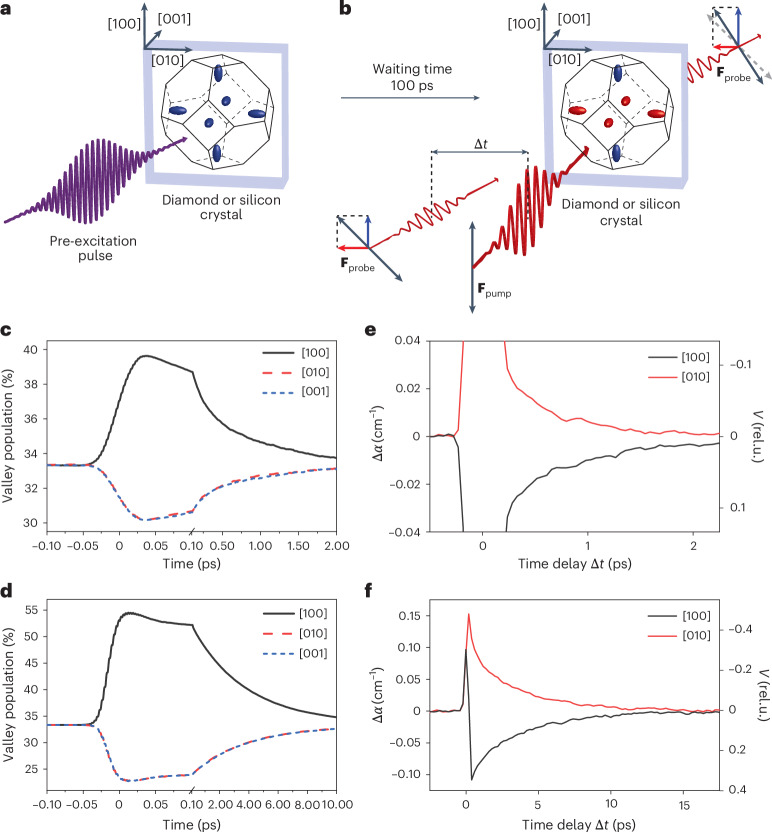
Generation and detection of the valley-polarized electron population in silicon and diamond. **a**, Electrons are excited by a resonant pre-excitation pulse and equally distributed to all conduction band valleys. Blue ellipsoids represent constant energy surfaces. **b**, After 100 ps, the linearly polarized infrared pump pulse with the electric field amplitude **F**_pump_ generates the valley-polarized electron distribution with a higher population in valleys with their principal axes parallel to the pump polarization (blue ellipsoids) than in the other valleys (red ellipsoids). The valley polarization is measured from the polarization anisotropy of free-carrier absorption of a linearly polarized probe pulse with polarization rotated by 45° (electric field **F**_probe_) incident on the sample in time delay Δ*t* with respect to the pump pulse. **c**,**d**, Time evolution of electron populations in the three inequivalent groups of valleys in silicon (**c**) and diamond (**d**) at room temperature calculated by Monte Carlo simulations. **e**,**f**, Measured polarization anisotropy of free-carrier absorption and the corresponding degree of valley polarization *V* (in relative units (rel.u.)) in silicon (**e**) with a pre-excited electron density *N* = 1.8 × 10^17^ cm^−3^ and diamond (**f**) with *N* = 6.4 × 10^16^ cm^−3^ at room temperature for two orientations of the pump polarization along the [100] direction (black curves) and [010] direction (red curves). The electric field amplitude of the pump pulse was 0.7 V nm^−1^ in silicon and 1.3 V nm^−1^ in diamond, in both the experiment and simulation. Source data

We show that valley polarization of the electrons in silicon and diamond crystals can be induced with an oscillating electric field of femtosecond infrared pulses. These two covalent semiconductors share the same double face-centred cubic structure, so that they have similar properties of the conduction band dispersion, although the bandgap energies differ substantially (1.12 eV in silicon and 5.5 eV in diamond). Both materials have an indirect band structure, and their conduction band dispersion *E*_c_(**k**) has six energy-degenerate minima close to the X point at the Brillouin zone edge in [100] and equivalent directions. The first Brillouin zone of silicon and diamond crystals is shown in Fig. 1a along with the surfaces of constant energy for the six degenerate valleys. In the parabolic approximation, the energy of an electron in the *i*th valley can be written as $${E}_\mathrm{c}^{\;(i)}({{\bf{k}}})=$$$${E}_{{{\rm{g}}}}+({\hslash }^{2}/2){\sum }_{j}{({k}_{j}-{k}_{j,0}^{(i)})}^{2}/{m}_{j}^{(i)}$$, where *E*_g_ is the bandgap energy, *k*_*j*_ denotes the individual components of the electron wavevector, $${k}_{j,0}^{(i)}$$ are the coordinates of the local band minimum and $${m}_{j}^{(i)}={\hslash }^{2}{\left[{\partial }^{2}{E}_\mathrm{c}^{\;(i)}({k}_{j})/\partial {k}_{j}^{2}\right]}^{-1}$$ is the electron effective mass. The electron effective mass tensor in each valley is strongly anisotropic, with the longitudinal effective mass *m*_l_ being approximately ×5 larger than the transverse effective mass *m*_t_ in both silicon and diamond^22,23^. There are three inequivalent groups of valleys with a large effective mass in crystallographic directions [100] (along **k**_*x*_), [010] (along **k**_*y*_) and [001] (along **k**_*z*_). When we apply an oscillating electric field in the [100] direction, an electron with the initial momentum **k**(0) = **k**_j,0_ (centre of one of the valleys) is accelerated in the **k**_*x*_ direction in the momentum space and its oscillations follow the pump field. Although the excursion of the electron in momentum space **k**_max_ = (*e***F**_0_)/(*ℏω*) does not depend on the effective mass, the average kinetic energy of an oscillating electron, $${U}_{{{\rm{p}}}}={(e| {{{\bf{F}}}}_{0}| )}^{2}/(4{\omega }^{2}{m}_{j}^{(i)})$$, differs depending on which valley the electron occupies due to the effective mass anisotropy. Here **F**_0_ is the field amplitude of the pump pulse, *e* is the electron charge and *ω* is the angular frequency of the pump field.

The electrons exchange energy and momentum with the crystal through their interaction with phonons. The mechanism of electron transfer between inequivalent groups of valleys is based on intervalley electron–phonon scattering (in the literature, this is referred to as f-scattering). To conserve momentum, such a transition requires the absorption or emission of a phonon with a large momentum. Specifically, the f-scattering between inequivalent valleys in silicon and diamond proceeds through the interaction of electrons with longitudinal and transverse acoustic and transverse optical phonons^24^. The equilibrium population of these modes at room temperature is low due to the relatively high energies of phonons with large momenta (Table 1). As a result, most of the intervalley transitions occur through phonon emission, which is possible only for electrons with a kinetic energy larger than the energy of the phonon involved in the scattering process. The dependence of the intervalley electron–phonon scattering rate on the electron energy combined with different values of the average kinetic energy of electrons in different groups of valleys during the interaction with the electric field of the infrared pump pulse leads to a higher probability of intervalley transitions of electrons from the valleys with a low effective mass *m*_t_ in the direction of the applied oscillating electric field (red ellipsoids in Fig. 1b) to the valleys with a large effective mass *m*_l_ (blue ellipsoids in Fig. 1b) than in the opposite direction. Although the unidirectional intervalley electron transitions occur only for the short duration of the infrared pulse, a substantial fraction of the electrons is transferred due to the high intervalley scattering rate of high-energy electrons of up to ~10^13^ s^−1^.

**Table 1:** Parameters of the Monte Carlo simulations

Quantity	Silicon	Diamond	Units
*ρ*	2.329	3.530	g cm^−^^3^
*m*_l_	0.92	1.56	*m*_0_
*m*_t_	0.19	0.28	*m*_0_
*D*_a_	9	8.7	eV
*u*_l_	8,430	17,520	m s^−1^
*D*_g_		16	10^8^ eV cm^−1^
*ℏΩ*_g_		160	meV
*D*_f1_	0.6		10^8^ eV cm^−1^
*ℏΩ*_f1_	19		meV
*D*_f2_	4	16	10^8^ eV cm^−1^
*ℏΩ*_f2_	47	134	meV
*D*_f3_	4	16	10^8^ eV cm^−1^
*ℏΩ*_f3_	59	148	meV
*F*_0_	0.7	1.3	V nm^−1^
*τ*_p_	40	40	fs

*D*_g_, *D*_f1_, *D*_f2_ and *D*_f3_ are deformation potentials related to g-type and f-type intervalley scattering processes, which are associated with phonons with frequencies *Ω*_g_, *Ω*_f1_, *Ω*_f2_ and *Ω*_f3_.

To verify that this mechanism can transfer electrons between inequivalent conduction band valleys within a few tens of femtoseconds, we solved the Boltzmann transport equation using a Monte Carlo approach closely following the treatment in ref. ^24^. In the simulations, we considered the intervalley scattering due to the interaction of electrons with acoustic and optical phonons and intravalley acoustic phonon scattering. The electron–phonon scattering is described in the framework of a deformation potential with the scattering rate dependent on the electron momentum and lattice temperature (details of the simulations are given in Methods). The calculated time evolution of electron populations in the three inequivalent groups of valleys with longitudinal effective mass along the [100], [010] or [001] directions during and after the interaction with the infrared femtosecond pump pulse is shown in Fig. 1c for silicon and in Fig. 1d for diamond. Both simulations assume room-temperature values for the crystal and pump pulse properties, which are applied in the following experiments. In silicon, up to 40% of the electrons are transferred to the two valleys with a longitudinal effective mass along the pump polarization after an interaction with an infrared pump pulse with a photon energy of 0.62 eV, a duration of 40 fs and a peak electric field *F*_0_ = 0.7 V nm^−1^. We can define the degree of valley polarization as *V* = (*N*_[100]_ − *N*_[010]_)/*N*, where *N*_[100]_ and *N*_[010]_ are the volume densities of electrons in valleys with the direction of the large effective mass along the [100] and [010] crystallographic directions, respectively, and *N* is the total pre-excited electron density in the conduction band. Under the conditions described above, the degree of valley polarization in silicon obtained from the numerical simulations is *V* ≈ 0.10. In diamond, the population of the two valleys with a large effective mass along the pump polarization direction reached 55% of the total electron population for a pump pulse with a field amplitude *F*_0_ = 1.3 V nm^−1^ corresponding to *V* ≈ 0.33. These values are comparable to the degree of valley polarization of 32% obtained from helicity-dependent photoluminescence measurements of two-dimensional transition-metal dichalcogenide MoS_2_ excited by circularly polarized light^25^. The calculated electron distributions in momentum space during and shortly after the interaction with the laser pulse is shown in Supplementary Videos 1 and 2 for both materials.

The valley polarization induced in silicon and diamond can be detected from the polarization anisotropy of the free-carrier absorption of a probe pulse. In the infrared spectral region, the difference between excited carrier absorption coefficients *α*_[100]_ and *α*_[010]_ for probe polarization components along the [100] and [010] directions, respectively, can be approximated using the Drude model as^26^:

1 $$\Delta \alpha ={\alpha }_{[100]}-{\alpha }_{[010]}=A\frac{\left({m}_{{{\rm{t}}}}-{m}_{{{\rm{l}}}}\right)\left({N}_{[100]}-{N}_{[010]}\right)}{{m}_{{{\rm{l}}}}{m}_{{{\rm{t}}}}},$$

where *A* is a constant (see Methods for details). Note that holes do not contribute to the measured Δ*α* because their effective mass tensor is symmetric with respect to rotation by 90°. The degree of valley polarization is directly proportional to the measured Δ*α* through equation (1) and can be expressed as *V* = Δ*αm*_l_*m*_t_/[*AN*(*m*_t_ − *m*_l_)].

The ultrafast optical generation and detection of the valley-polarized electron population in both studied materials was experimentally demonstrated using the scheme shown in Fig. 1a,b (a detailed layout of the experimental set-up is shown in Supplementary Fig. 1). First, the electrons and holes were excited by a pre-excitation pulse (Fig. 1a). To excite the electrons with a homogeneous distribution along the light propagation direction, we used indirect single-photon excitation in silicon (photon energy 1.2 eV) and two-photon excitation in diamond (photon energy 3.6 eV). Several picoseconds after the optical excitation, the electrons relaxed to the band minima by electron–phonon scattering and became isotropically distributed to the six degenerate conduction band valleys (blue ellipsoids in Fig. 1a). Then, 100 ps after the photo-excitation, the crystal was illuminated by an infrared pump pulse with linear polarization along the [100] or [010] direction, which induced the valley polarization. The waiting time of 100 ps was selected to ensure that the electronic system had already relaxed to the lattice temperature when the pump pulse arrived at the sample. Note that this time delay is substantially shorter than the lifetime of excited carriers in both materials^27,28^. The anisotropy of the [100] and [010] polarization components of the free-carrier absorption was measured using a probe pulse with linear polarization rotated by 45° with respect to the polarization of the pump (along the [$\bar{1}$10] direction), which was incident on the sample Δ*t* after the pump pulse. Both the pump and probe pulses had a photon energy of 0.62 eV (central wavelength of 2,000 nm) and a pulse duration of 40 fs. The peak electric field of the pump pulse reached 1.3 V nm^−1^ in the experiments performed in silicon and 1.5 V nm^−1^ in diamond (details of the experimental set-up are discussed in Methods).

The measured polarization anisotropy of free-carrier absorption Δ*α* at room temperature is shown in Fig. 1e,f for both studied materials with the polarization of the pump along the [100] (black curves) and [010] (red curves) directions. The different signs of Δ*α* for the two orthogonal directions of pump polarization confirm that the electrons were transferred preferentially to the valleys with the longitudinal effective mass along the direction of pump polarization. In the following experiments, the difference between the measured Δ*α* with pump polarization along [100] and [010] was used to remove any possible experimental artefacts that could have influenced the measurement of the polarization anisotropy of free-carrier absorption. The fast signal at Δ*t* = 0 ps was caused by the coherent interaction of the pump and probe pulses. The valley-polarized electron population was proportional to the slow exponentially decaying component of Δ*α*.

**Extended Data Fig. 1:**
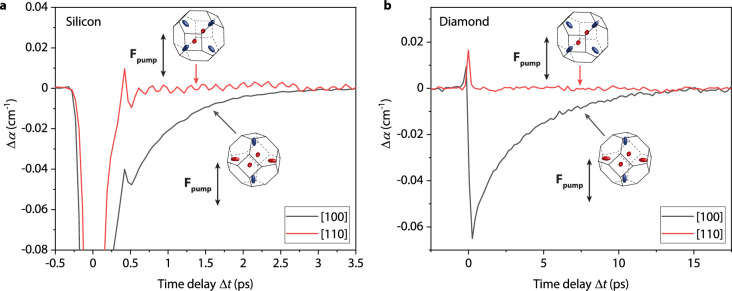
Polarization anisotropy of free-carrier absorption in silicon and diamond. Measured difference between the polarization components of free-carrier absorption parallel and perpendicular to the pump field Δ*α* in **a** silicon and **b** diamond for sample orientation with pump field along [100] direction (black curves) and Δ*α*_[110]_ for [110] direction (red curves). The oscillations observed in the signal with pump polarization along [110] in silicon are due to impulsive excitation of coherent optical phonons with energy of 65 meV, which is smaller than the spectral width of the pump pulse, via stimulated Raman scattering. This process is not allowed with pump polarization along [100] due to symmetry reasons. Phonon oscillations are not present in diamond because the optical phonon energy of 160 meV is larger than the bandwidth of the pump pulse. Insets: Brillouin zone of silicon and diamond with the six conduction band valleys for the configuration with pump along [100] direction (lower insets) and [110] direction (upper insets). Blue ellipsoids correspond to the valleys with higher electron population after interacting with the pump pulse while red ellipsoids show valleys with lower electron population. Source data

To verify the origin of the non-zero Δ*α* in the valley-polarized electron population, we rotated both crystals by 45° such that the pump polarization was along [110] or [1$\bar{1}$0] (Extended Data Fig. 1). We did not observe in this configuration any decaying Δ*α*_[110]_ = *α*_[110]_ − $\alpha_{[1\bar{1}0]}$ because the four valleys with a longitudinal effective mass perpendicular to [001] (blue ellipsoids in the upper insets of Extended Data Fig. 1a,b) were populated isotropically for pump polarization along [110] and [1$\bar{1}$0].

**Extended Data Fig. 2:**
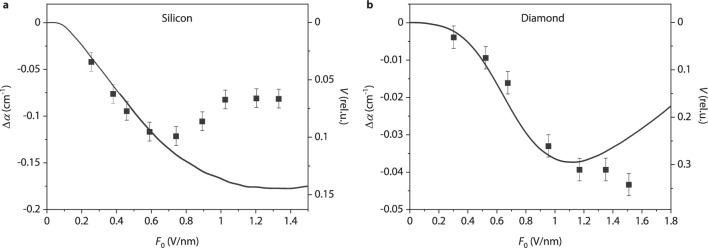
Dependence of polarization anisotropy of free-carrier absorption in silicon and diamond on the electric field amplitude of the pump pulse. Measured difference between the polarization components of free-carrier absorption parallel and perpendicular to the pump field Δ*α* and the associated degree of valley polarization *V* in **a** silicon and **b** diamond (black squares) at room temperature as a function of the electric field amplitude of the pump pulse *F*_0_. The data represent the amplitude of the single-exponential decay fit of the measured dynamics of Δ*α*. The experimental data are compared to the calculated Δ*α* (black curve) obtained from Monte Carlo simulations. Δ*α* is calculated from the simulated electron populations in individual valleys in the time delay of Δ*t*=100 fs using Drude model with the electron density of *N*_Si_ = 7.5 × 10^17^ cm^−3^ in silicon and *N*_C_ = 2.6 × 10^16^ cm^−3^ in diamond. The values of the average electron scattering time in the two materials used in the Drude model are *τ*_Si_ = 90 fs and *τ*_C_ = 30 fs, respectively. The error bars represent the estimated total measurement uncertainty. The main source of the uncertainty is the long term fluctuations of experimental conditions (power and pointing stability of the laser system). Source data

**Extended Data Fig. 3:**
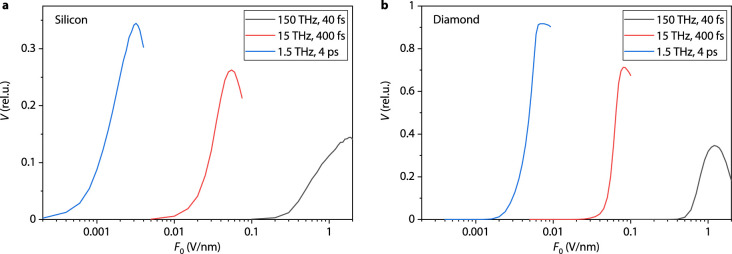
Numerical simulations of the degree of valley polarization induced in silicon and diamond at temperature of 77 K using pump pulses with different parameters. The data show the calculated degree of valley polarization *V* as a function of the peak electric field amplitude of the pump pulse in **a** silicon and **b** diamond for three different sets of pulse parameters, namely central photon energy of 0.62 eV (carrier frequency *f*=150 THz) and pulse duration 40 fs (black curves, pulse parameters applied in the experiments presented in this work), central photon energy of 62 meV (*f*=15 THz) and pulse duration of 400 fs (red curves) and central photon energy of 6.2 meV (*f*=1.5 THz) and pulse duration of 4 ps (blue curves). Source data

To generate the valley-polarized electron population, the amplitude of the electric field of the pump pulse had to be adjusted such that the maximum of the instantaneous energy of electrons in the valleys with a low effective mass became higher than the energy of the phonon required for intervalley scattering whereas the energy of the electrons from the valleys with a large effective mass had to stay below the phonon energy. The measured dependence of the slow component of Δ*α* and the corresponding degree of valley polarization of electrons on the electric field amplitude of the pump pulse is shown in Extended Data Fig. 2 for both materials and compared to numerical simulations. The degree of the induced valley polarization saturated and even decreased for an excessively large pump field. The difference between the measured data and the numerical calculations in silicon at field amplitudes *F*_0_ > 0.7 V nm^−1^ occurred because the photon energy of the pump was close to the silicon bandgap energy. The non-resonant pump induced transitions to higher conduction bands, from which the electrons relaxed and became isotropically distributed to the six valleys. This effect decreased the degree of valley polarization at pump field *F*_0_ > 0.7 V nm^−1^. The discrepancy between the measured and calculated degree of valley polarization in diamond at field amplitude *F*_0_ > 1.3 V nm^−1^ was probably due to the band non-parabolicity, which was not taken into account in the numerical simulations. The generated degree of valley polarization could be optimized by modifying the photon energy, duration and peak field of the pump pulses. Numerical simulations at a crystal temperature of 77 K, as shown in Extended Data Fig. 3, indicate that on decreasing the frequency of the pump pulse to the terahertz region, the maximal degree of valley polarization reached *V* = 0.35 in silicon and *V* = 0.92 in diamond. Note that the amplitude of the oscillating field required to generate the valley-polarized electron population was about four orders of magnitude higher than the amplitude of the d.c. field required to reach valley polarization in diamond at low temperatures^18^ and it strongly depends on the frequency of the pump pulse (Extended Data Fig. 3).

**Fig. 2:**
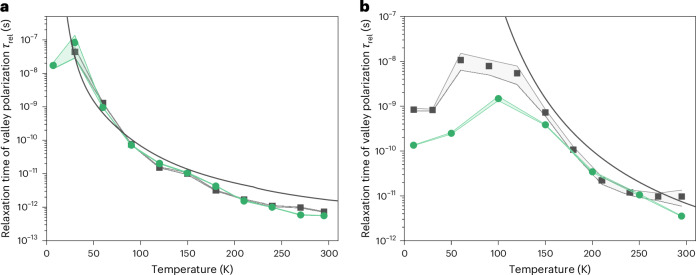
Relaxation time of valley polarization in silicon and diamond crystals as a function of lattice temperature. **a**,**b**, Measured relaxation time of valley polarization *τ*_rel_ in silicon (**a**) and diamond (**b**) compared with the intervalley scattering time obtained from the numerical solution of the Boltzmann transport equation using a Monte Carlo approach (solid curve). The experimental points represent the decay time obtained by fitting the experimental data for Δ*α* averaged over 20 temporal scans per point by a single-exponential decay function. The shaded regions represent the standard deviation of the exponential decay time obtained by fitting the measured dynamics of Δ*α*. The pre-excited electron density is *N* = 1.8 × 10^17^ cm^−3^ (black squares) and *N* = 1.5 × 10^18^ cm^−3^ (green circles) for the data in **a** and *N* = 4.4 × 10^15^ cm^−3^ (black squares) and *N* = 6.4 × 10^16^ cm^−3^ (green circles) for the data in **b**. Source data

Compared to previous experiments investigating the valley polarization of electrons in silicon^12,29^ or diamond^18^, our method offers femtosecond-time resolution, which allowed us to directly characterize the relaxation dynamics of valley polarization due to intervalley electron–phonon scattering in both crystals studied. The measured relaxation times of valley polarization are shown in Fig. 2a,b as a function of sample temperature for electron densities *N* = 1.8 × 10^17^ cm^−3^ (black squares in Fig. 2a) and *N* = 1.5 × 10^18^ cm^−3^ (green circles in Fig. 2a) in silicon and *N* = 4.4 × 10^15^ cm^−3^ (black squares in Fig. 2b) and *N* = 6.4 × 10^16^ cm^−3^ (green circles in Fig. 2b) in diamond. The carrier density was controlled by the fluence of the pre-excitation pulse. At the lowest carrier densities used in our experiments, the measured relaxation times of valley polarization at room temperature were *τ*_rel_ = 730 fs in silicon and *τ*_rel_ = 9.7 ps in diamond. The former value can be compared to the L → X intervalley scattering time of 180 fs reported for electrons in silicon in ref. ^21^. For both materials, the relaxation time of valley polarization increased with decreasing temperature, in agreement with numerical calculations of the intervalley scattering time (solid curves in Fig. 2a,b; for details, see Methods), reaching *τ*_rel_ ≈ 80 ns in silicon and *τ*_rel_ ≈ 10 ns in diamond. The valley-polarized population of electrons thus persisted in these materials at low temperatures for much longer times than in two-dimensional transition-metal dichalcogenides, for which intervalley scattering times of only a few picoseconds have been reported^30^. Although the measured relaxation time depended strongly on the excited carrier density in the diamond crystal at low temperatures, the dynamics in silicon practically did not change on changing the pre-excited electron density. This was due to Coulomb interactions and many-body phenomena, which led to the formation of exciton molecules and electron–hole droplets with different critical temperatures in diamond *T*_c_ = 165 K (refs. ^31,32^) and silicon *T*_c_ = 27 K (refs. ^33,34^). Below these temperatures, electron–hole condensation led to Auger recombination. In this non-radiative process, an electron–hole pair recombines and transfers its energy to another quasiparticle (electron or hole). Compared to previous experiments on diamond in which a valley relaxation time of 300 ns was reported at 77 K with a very low excited carrier density of below *N* = 10^10^ cm^−3^ (ref. ^18^), the relaxation of valley polarization at low temperatures was in our case (excited carrier density *N* = 10^15^ to 10^17^ cm^−3^) influenced by faster carrier recombination and by the higher intervalley scattering rate of electrons, which increased due to the excess energy obtained during the Auger process.

Even at moderate excited electron densities of the order of *N* = 10^13^ to 10^15^ cm^−3^, the measured relaxation time of valley polarization in diamond is influenced by Coulomb interactions. Owing to the high binding energy of Wannier excitons of 80 meV (ref. ^35^), most of the excited carriers are bound in the excitonic state at low temperatures (the balance between excitons and free electrons and holes can be described using the Saha equation^36,37^). The excitons influence the generation and relaxation of valley polarization through an increased deformation potential of the exciton state compared to the single electron state.

**Fig. 3:**
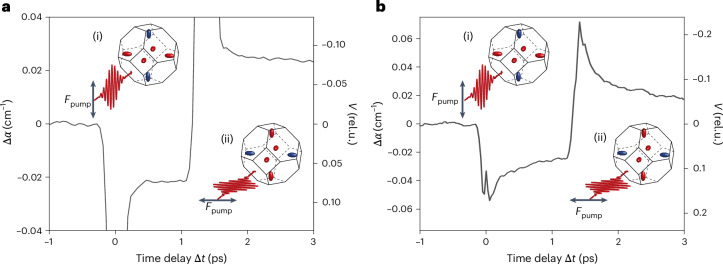
Switching the valley polarization of electrons in silicon and diamond at terahertz frequencies. **a**,**b**, Valley-polarized electron population is generated in silicon at temperature 7 K (**a**) and in diamond at room temperature (295 K) (**b**) using a pump pulse with linear polarization along the [100] direction (insets (i) in **a** and **b**). After 1.4 ps, a second pump pulse with linear polarization along the [010] direction (insets (ii) in **a** and **b**) switches the direction of valley polarization, which manifests itself as a change of sign of the measured Δ*α* of the probe pulse, which is directly proportional to the degree of valley polarization *V*. Source data

The possibility of switching the valley polarization on femtosecond timescales could be used in valleytronic devices working at terahertz frequencies. To demonstrate the ultrafast capabilities of our method for generating, switching and reading out valley polarization of electrons, we separately illuminated the silicon and diamond crystals by a pair of infrared pump pulses separated in time. The first pump pulse was linearly polarized parallel in the [100] direction and generated the valley polarization. Most of the electrons were in the valleys with their longitudinal effective mass along [100] leading to a decrease of the measured Δ*α*. The second pump pulse arrived at the sample after a time delay of 1.4 ps with respect to the first pump and was linearly polarized along the [010] direction (see Methods for details of pulse pair generation). It rotated the direction of valley polarization and, thus, the sign of the measured Δ*α* (see Fig. 3a for the measurements in silicon at temperature 7 K and Fig. 3b for the measurements in diamond at room temperature). The time needed for valley polarization switching and its observation was ultimately limited by the duration of the pump and probe pulses and by electron relaxation in the valleys. In silicon, the time resolution was also limited by the coherent interaction of the pump and probe pulses, which induced two-photon transitions to higher conduction bands (decrease of Δ*α* over time Δ*t* = 0 ps and its increase over Δ*t* = 1.4 ps). We clearly reached subpicosecond switching of the valley polarization of the electrons in both materials.

There is a large number of materials in which the valley polarization of electrons has never been observed due to the lack of an ultrafast method that would allow us to induce and detect the anisotropic electron population in momentum space before it relaxes back to the isotropic state. Owing to its non-resonant nature, the technique presented here can be applied to generate a valley-polarized electron population in such materials with only two requirements: (1) the effective mass tensor of the electrons must have different anisotropy in inequivalent valleys and (2) the intervalley scattering rate must depend on the electron energy in the band. However, these two conditions are generally valid for any semiconductor or dielectric material with several degenerate minima of the conduction band. The principles demonstrated here thus open the field of valleytronics to a wide range of crystals and possibly also to nanocrystalline materials.

## Methods

### Experimental set-up

The infrared pump and probe pulses with a photon energy of 0.62 eV, duration of 40 fs and repetition rate of 25 kHz were generated in a non-collinear optical parametric amplifier-difference frequency generation set-up pumped by a ytterbium femtosecond laser (Pharos SP, Light Conversion)^38^. Pulses with a photon energy of 1.2 eV and duration of 170 fs (fundamental output from the laser) were used as pre-excitation pulses for the experiments on silicon. The spot size (1/e^2^ radius, where e is the Euler number) on the sample surface was *w*_NIR_ = 73 μm. For experiments on diamond, we used pre-excitation pulses with a photon energy of 3.6 eV corresponding to the third harmonic frequency of the fundamental laser output. The beam was focused to a spot size *w*_UV_ = 57 μm. The peak excited carrier density was calculated from the measured absorbance of the pre-excitation pulses and the known area of the laser foci. The time delays between the pre-excitation, the pump and the probe pulses were controlled using two independent optical delay lines.

The infrared pump and probe pulses were focused onto the sample using an off-axis parabolic mirror with a focal distance *f* = 100 mm. The spot size of both the pump and probe beams was *w*_0_ = 26 μm. The direction of polarization of the pump pulse was controlled by a broadband half-wave plate. The sample was placed in a closed-cycle helium cryostat, which allowed us to control its temperature in the range 7–295 K. The polarization anisotropy of free-carrier absorption was measured by splitting the probe with linear polarization rotated by ±45° with respect to the polarization of the pump pulse using a Glan-laser polarizer. The two polarization components of the probe—parallel and perpendicular to the polarization of the pump pulse—were detected by InGaAs photodiodes, and the difference signal was measured by a lock-in amplifier. The amplifier was locked to the frequency of an optical chopper, which was placed into the pre-excitation beam. This configuration was sensitive only to the polarization anisotropy of free-carrier absorption induced by the pump on the distribution of pre-excited carriers. Assuming that the relative change of the sample transmission induced by excited carriers was small, the absolute value of the polarization anisotropy of free-carrier absorption Δ*α* was calculated from the measured differential signal *S*_A−B_ as Δ*α* = *S*_A−B_/(*S*_probe_*d*), where *S*_probe_ is the signal corresponding to the power of the transmitted probe pulse obtained by inserting the optical chopper into the probe beam and *d* is the sample thickness.

The two time-separated pump pulses with orthogonal polarizations used in the switching experiments (results shown in Fig. 3) were generated by sending a linearly polarized pump pulse with polarization rotated by 45° through an optically anisotropic crystal of beta-barium borate. This negative uniaxial anisotropic material introduced a group delay of 1.4 ps between the extraordinary and ordinary polarization components of the pump pulse polarized along the [100] and [010] crystallographic directions of silicon and diamond crystals.

### Numerical solution of the Boltzmann transport equation using a Monte Carlo approach

To theoretically describe the electron dynamics in momentum space during and after illumination of the silicon and diamond crystals with the infrared pump pulse, we numerically solved the Boltzmann transport equation using a Monte Carlo approach following the treatment in ref. ^24^. This allowed us to include both the anisotropic effective mass tensor of electrons in individual valleys and the energy- and momentum-dependent intra- and intervalley scattering mechanisms. Note that we solved only the equations for electrons because valence bands have their maxima at the Γ point of the first Brillouin zone and because the hole effective mass tensor is symmetric with respect to rotation by 90° in all valence bands (the effective mass of holes has the same value along the [100] and [010] directions). Assuming a homogeneous distribution of electrons in real space, the time evolution of the momentum-dependent distribution function *f*(**k**, *t*) can be written as:

2 $$\frac{\partial f({{\bf{k}}},t)}{\partial t}-\frac{e{\bf{F}}(t)}{\hslash }{\nabla }_{{{\bf{k}}}}\;f({{\bf{k}}},t)={\left.\frac{\partial f({{\bf{k}}},t)}{\partial t}\right\vert }_{{{\rm{coll}}}},$$

where ${\bf{F}}(t)={{{\bf{F}}}}_{0}\exp \left[-(2\ln 2{(t-{t}_{0})}^{2})/{\tau }_{{{\rm{p}}}}^{2}\right]\cos \left(\omega t\right)$ is the time-dependent electric field of the pump pulse with amplitude **F**_0_, angular frequency *ω* = 2π*f* with *f* being the central frequency of the pulse spectrum, *τ*_p_ is the full-width at half-maximum duration and *e* is the elementary charge. The right-hand side represents the change of the distribution function due to collisions. The Monte Carlo approach is based on solving the equation of motion for individual randomly chosen electrons from the initial distribution *f*(**k**, 0), where the collision term is expressed using a probability of an electron scattering per unit volume and time. The collision term has a characteristic dependence on the electron energy and momentum for each type of scattering process. In our simulations, we assumed a parabolic conduction band with six degenerate minima (valleys). Each valley had an anisotropic effective mass tensor. The longitudinal and transverse effective masses are given in Table 1. Note that the parabolic approximation is sufficient to describe the generation and relaxation of valley polarization for our experimental parameters. The maximum kinetic energy that the electrons acquired during the oscillatory motion driven by the pump laser field was 250 meV in silicon (*F*_0_ = 0.7 V nm^−1^) and 0.6 eV in diamond (*F*_0_ = 1.3 V nm^−1^), which were much less than the depth of the valleys. The band non-parabolicity mainly influenced the coherent nonlinear response of the electrons to the driving pump field. The intervalley scattering rates did not change substantially when taking the non-parabolicity into account.

To describe the electron collisions, we assumed several inelastic electron–phonon scattering mechanisms, namely the intravalley and intervalley electron scattering on acoustic and optical phonons. The probability per unit time of an intravalley electron scattering on acoustic phonons from the initial state with momentum **k** to the final state with momentum **k**′ is

3 $${P}_{{{\rm{a}}}}({{\bf{k}}},{{{\bf{k}}}}^{{\prime} })=\frac{\uppi q{D}_{{{\rm{a}}}}^{2}}{V\rho {u}_\mathrm{l}}\left[{\left(\operatorname{e}^{{\hslash q{u}_{{{\rm{l}}}}}/{{k}_{{{\rm{B}}}}T}}-1\right)}^{-1}+\frac{1}{2}\pm \frac{1}{2}\right]\delta \left({E}_\mathrm{c}({{{\bf{k}}}}^{{\prime} })-{E}_\mathrm{c}({{\bf{k}}})\pm \hslash q{u}_{{{\rm{l}}}}\right),$$

where *q* is the length of the phonon wavevector, *D*_a_ is the deformation potential describing the inelastic interaction of electrons with acoustic phonons, *V* is the crystal volume, *ρ* is the material density, *u*_l_ is the propagation velocity of longitudinal acoustic phonons (speed of sound) in the material, *k*_B_ is the Boltzmann constant and *T* is temperature. The intervalley electron scattering rate and the intravalley scattering rate for optical phonons was calculated as:

4 $$\begin{array}{l}{P}_{{{\rm{f}}}({{\rm{g}}})}({{\bf{k}}},{{{\bf{k}}}}^{{\prime} })=\displaystyle\frac{g\,\uppi {D}_{{{\rm{f}}}({{\rm{g}}})}^{2}}{V\rho {\varOmega }_{{{\rm{f}}}({{\rm{g}}})}}\left[{\left(\operatorname{e}^{{\hslash {\varOmega }_{{{\rm{f}}}({{\rm{g}}})}}/{{k}_{{{\rm{B}}}}T}}-1\right)}^{-1}+\frac{1}{2}\pm \displaystyle\frac{1}{2}\right]\\\qquad\qquad\quad\delta\,\left({E}_\mathrm{c}({{{\bf{k}}}}^{{\prime} })-{E}_\mathrm{c}({{\bf{k}}})\pm \hslash {\varOmega }_{{{\rm{f}}}({{\rm{g}}})}\right),\end{array}$$

where *D*_f(g)_ is the deformation potential describing the scattering of electrons at high-energy optical or acoustic phonons leading to f-type (g-type) intervalley scattering, *g* is the number of equivalent final valleys (*g* = 4 for f-type intervalley optical phonon scattering to inequivalent valleys and *g* = 2 for g-type optical phonon scattering to equivalent valleys) and *Ω*_f(g)_ is the frequency of the phonon involved in the intervalley scattering. In the simulations of electron dynamics in silicon, we took into account f-type intervalley scattering at transverse and longitudinal acoustic phonons and transverse optical phonons. In the simulations of electron dynamics in diamond, we took into account g-type scattering at longitudinal optical phonons and f-type scattering at longitudinal acoustic and transverse optical phonons^24^. The parameters used in the simulations are summarized in Table 1. All the parameters, except the intervalley deformation potentials, are from ref. ^20^. For the deformation potentials, various values have been reported in literature for both materials (for example, refs. ^18,20,24,39^ and references therein). The acoustic deformation potential and the energies of phonons participating in f-type and g-type intervalley scattering in both materials are from ref. ^24^. The deformation potential associated with the intervalley transitions from ref. ^24^ lead to excessively long intervalley scattering times. For this reason, we used deformation potentials as fitting parameters. The final values were in close agreement with the values reported in refs. ^18,39^.

### Polarization anisotropy of free-carrier absorption as described by the Drude model

The free-carrier absorption of infrared light due to free electrons and holes excited in the conduction and valence bands and relaxed to thermal equilibrium with the lattice can be described using the Drude model, which is a satisfactory approximation in the infrared spectral region. Assuming that the scattering rate was much lower than the frequency of the incident light and that the imaginary part of the dielectric function was small, the absorption induced by free electrons in a crystal for the isotropic conduction band can be approximated as^26^:

5 $${\alpha }_{{{\rm{Drude}}}}=\frac{{e}^{2}N}{{\varepsilon }_{0}{m}^{* }c{n}_{0}{\omega }^{2}\tau }=A\frac{N}{{m}^{* }},$$

where *N* is the electron density, *ε*_0_ is the dielectric function of vacuum, m^*^ is the electron (hole) effective mass, *c* is the speed of light, *n*_0_ is the refractive index of the material, *ω* is the frequency of light, *τ* is the electron scattering time and *A* is the constant used in equation (1). Equation (5) has to be modified to describe the total free-carrier absorption including different populations of electrons in inequivalent valleys, the anisotropic effective mass of electrons and the presence of holes. The total free-carrier absorption coefficients in silicon and diamond for the infrared light linearly polarized along the [100] and [010] directions can be written as:

6 $$\begin{aligned}{\alpha }_{[100]}&=\frac{{e}^{2}}{{\varepsilon }_{0}c{n}_{0}{\omega }^{2}\tau }\left(\frac{{N}_{[100]}}{{m}_{{{\rm{l}}}}}+\frac{{N}_{[010]}+{N}_{[001]}}{{m}_{{{\rm{t}}}}}+\frac{{N}_{{{\rm{h}}}}}{{m}_{{{\rm{h}}}}}\right),\\ {\alpha }_{[010]}&=\frac{{e}^{2}}{{\varepsilon }_{0}c{n}_{0}{\omega }^{2}\tau }\left(\frac{{N}_{[010]}}{{m}_{{{\rm{l}}}}}+\frac{{N}_{[100]}+{N}_{[001]}}{{m}_{{{\rm{t}}}}}+\frac{{N}_{{{\rm{h}}}}}{{m}_{{{\rm{h}}}}}\right),\end{aligned}$$

where *N*_[100]_, *N*_[010]_ and *N*_[001]_ are the real-space electron densities in the valleys with principal axes along the [100], [010] and [001] directions, *N*_h_ = (*N*_[100]_ + *N*_[010]_ + *N*_[001]_) is the density of holes and *m*_h_ is the effective mass of holes (an average value over all bands when taking into account their occupation). We assumed that the average carrier scattering time *τ* is isotropic and constant for electrons and holes. Note that the Drude model has been proven to describe the dielectric response of silicon in the low-frequency spectral region under non-equilibrium conditions^40^. The polarization anisotropy of free-carrier absorption (the result of equation (1)) was calculated as Δ*α* = *α*_[100]_ − *α*_[010]_. The average electron scattering times *τ* used in the Drude model to calculate the absolute values of the polarization anisotropy of free-carrier absorption were *τ*_Si_ = 90 fs in silicon and *τ*_C_ = 30 fs in diamond.

The experimental value of Δ*α* was determined from the measured absorption coefficients of the individual polarization components of the probe beam:

7 $${\alpha }_{[100]([010])}=-\frac{\Delta {T\,}^{[100]([010])}}{{T}_{0}d},$$

where Δ*T*^[100]([010])^ corresponds to the change of transmission of the probe beam polarized along the [100] ([010]) direction induced by the excited charge carriers, *T*_0_ is the probe transmission in an unexcited sample and *d* is the sample thickness. We assumed that the free-carrier absorption was weak and that Δ*T* ≪ *T*_0_.

### Silicon and diamond crystals

The monocrystalline silicon sample used in this study was purchased from MicroChemicals GmbH. It is an intrinsic crystal without doping with a low concentration of impurities and a high resistivity of *ρ* > 20,000 Ω cm. The crystal was grown by vertical zone melting. The sample had a thickness of *d* = 525 μm. The diamond crystal was manufactured by ElementSix using chemical vapour deposition. It had a low amount of impurities (<1 ppb of boron and <5 ppb of nitrogen as specified by the manufacturer). The crystal thickness was *d* = 500 μm. Both sides of both samples were polished, and the surface normal was oriented in the [001] direction.

## Online content

Any methods, additional references, Nature Portfolio reporting summaries, source data, extended data, supplementary information, acknowledgements, peer review information; details of author contributions and competing interests; and statements of data and code availability are available at 10.1038/s41567-025-02862-4.

## Supplementary information

Supplementary Information Supplementary Fig. 1.

Supplementary Video 1 Monte Carlo simulation of electron dynamics during the interaction with the electric field of the infrared pump pulse in the first Brillouin zone of silicon at room temperature. The pump pulse with linear polarization along the [100] (*k*_*x*_) direction accelerated the electron population in momentum space. The electrons that initially were in valleys with a large effective mass in the direction of the pump field are represented by blue dots. Electrons from valleys with a low effective mass are represented by red dots. During the interaction with the pump field, the lighter electrons mostly underwent intervalley scattering whereas the heavier electrons preferentially remained in the same valley.

Supplementary Video 2 Monte Carlo simulation of electron dynamics during the interaction with the electric field of the infrared pump pulse in the first Brillouin zone of diamond at room temperature. The pump pulse with linear polarization along the [100] (*k*_*x*_) direction accelerated the electron population in momentum space. The electrons that initially were in valleys with a large effective mass in the direction of the pump field are represented by blue dots. Electrons from valleys with a low effective mass are represented by red dots. During the interaction with the pump field, the lighter electrons mostly underwent intervalley scattering whereas the heavier electrons preferentially remained in the same valley.

## Source data

Source Data Fig. 1c Source data for graph.

Source Data Fig. 1d Source data for graph.

Source Data Fig. 1e Source data for graph.

Source Data Fig. 1f Source data for graph.

Source Data Fig. 2a Source data for graph.

Source Data Fig. 2b Source data for graph.

Source Data Fig. 3 Source data for graph.

Source Data Extended Data Fig. 1a Source data for graph.

Source Data Extended Data Fig. 1b Source data for graph.

Source Data Extended Data Fig. 2a Source data for graph.

Source Data Extended Data Fig. 2b Source data for graph.

Source Data Extended Data Fig. 3a Source data for graph.

Source Data Extended Data Fig. 3b Source data for graph.

## Data Availability

All the data that support the plots and the other findings of this study are available via Zenodo at 10.5281/zenodo.15004810 (ref. ^41^). Source data are provided with this paper.

## Code availability

All the computational codes that were used to generate the data presented in this study are available from the corresponding author upon reasonable request.
