# Supplementary material for: Ultrafast room-temperature valley manipulation in silicon and diamond
Source: Nat Phys. 2025 Apr 14;21(6):947–52. doi: 10.1038/s41567-025-02862-4 (PMC12176610; doi:10.1038/s41567-025-02862-4)
Supplement: Supplementary file 1 — Supplementary Fig. 1. [file 41567_2025_2862_MOESM1_ESM.pdf]

nature physics

Supplementary information https://doi.org/10.1038/s41567-025-02862-4

Ultrafast room-temperature valley manipulation in silicon and diamond

In the format provided by the authors and unedited

Contents

Supplementary Figure 1 **2**

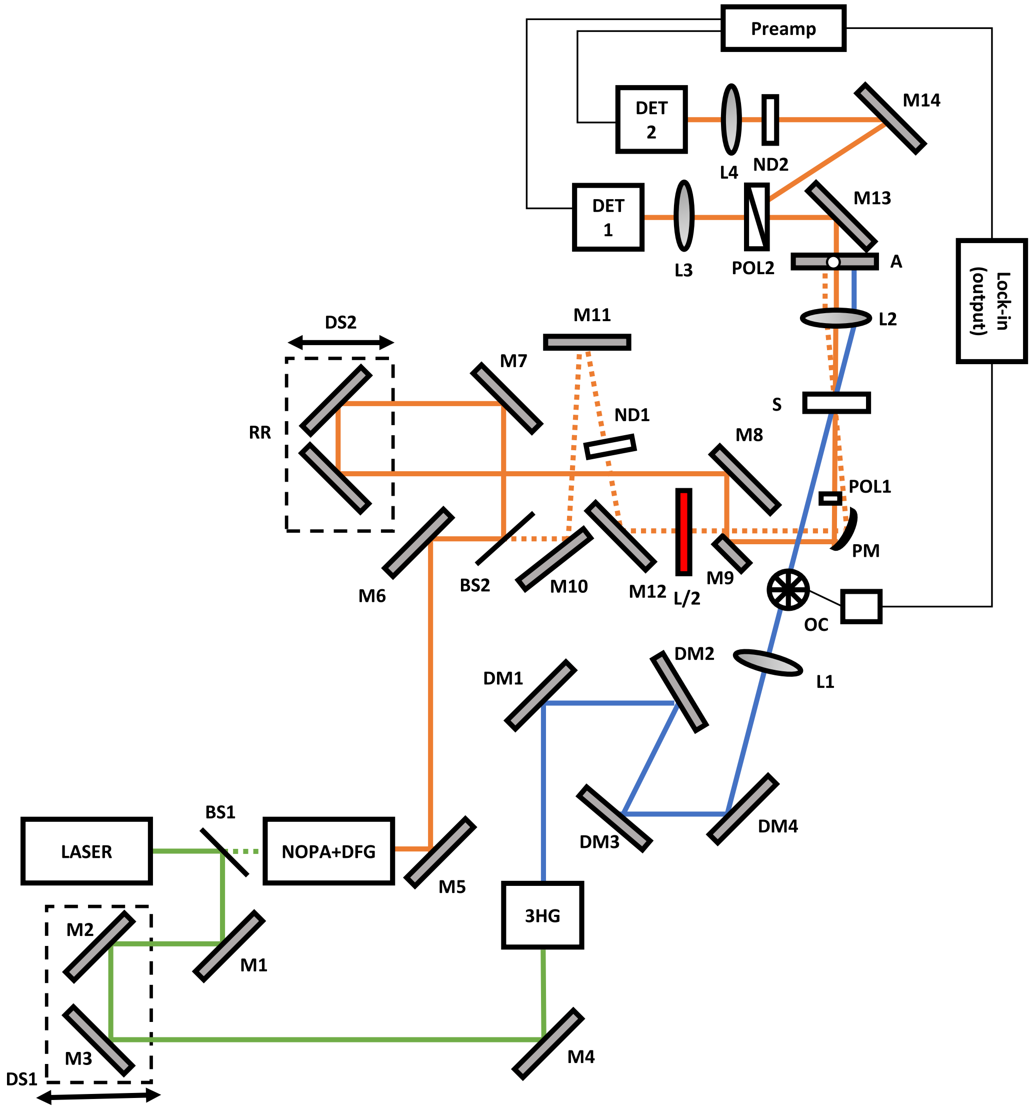

Supplementary Figure 1: Detailed layout of the experimental setup. LASER - femtosecond laser system Pharos SP 6W (Light Conversion), NOPA+DFG - noncolinear optical parametric amplifier with subsequent difference frequency generation setup, where pump and probe pulses are generated, 3HG - third harmonic generation (used only during the experiments in diamond), BS1-2 - beam-splitter, M1-14 - silver mirror, DS1-2 - delay stage, DM1-4 - dielectric mirror, A - aperture, ND1-2 - neutral density filter, L1-4 - optical lens, OC - optical chopper, S - sample, RR - retroreflector, PM - parabolic mirror, POL1-2 - polarizer, L/2 - half-wave plate, DET1-2 - detector, Preamp - preamplifier, Lock-in - lock-in amplifier.
